# Clinical assessment of liver metabolism during hypothermic oxygenated machine perfusion using microdialysis

**DOI:** 10.1111/aor.14066

**Published:** 2021-09-21

**Authors:** Damiano Patrono, Dorotea Roggio, Anna Teresa Mazzeo, Giorgia Catalano, Elena Mazza, Giorgia Rizza, Alessandro Gambella, Federica Rigo, Nicola Leone, Vincenzo Elia, Daniele Dondossola, Caterina Lonati, Vito Fanelli, Renato Romagnoli

**Affiliations:** ^1^ General Surgery 2U ‐ Liver Transplant Unit, Department of Surgical Sciences, A.O.U. Città della Salute e della Scienza di Torino University of Turin Turin Italy; ^2^ Department of Molecular Biotechnology and Health Sciences University of Turin Turin Italy; ^3^ Anaesthesia, Critical Care and Emergency, A.O.U. Department of Surgical Sciences, Città della Salute e della Scienza di Torino University of Turin Turin Italy; ^4^ Anesthesia and Intensive Care, Department of Adult and Pediatric Pathology University of Messina Messina Italy; ^5^ Pathology Unit, Department of Medical Sciences University of Turin Turin Italy; ^6^ General and Liver Transplant Surgery Unit Fondazione IRCCS Ca’ Granda Ospedale Maggiore Policlinico Milan Italy; ^7^ Department of Pathophysiology and Transplantation Università degli Studi of Milan Milan Italy; ^8^ Center for Preclinical Research Fondazione IRCCS Ca’ Granda Ospedale Maggiore Policlinico Milan Italy

**Keywords:** extracellular fluid, flavin mononucleotide, liver metabolism, liver viability assessment, machine perfusion, microdialysis

## Abstract

**Background:**

While growing evidence supports the use of hypothermic oxygenated machine perfusion (HOPE) in liver transplantation, its effects on liver metabolism are still incompletely understood.

**Methods:**

To assess liver metabolism during HOPE using microdialysis (MD), we conducted an open‐label, observational pilot study on 10 consecutive grafts treated with dual‐HOPE (D‐HOPE). Microdialysate and perfusate levels of glucose, lactate, pyruvate, glutamate, and flavin mononucleotide (FMN) were measured during back table preparation and D‐HOPE and correlated to graft function and patient outcome.

**Results:**

Median (IQR) MD and D‐HOPE time was 228 (210, 245) and 116 (103, 143) min. Three grafts developed early allograft dysfunction (EAD), with one requiring retransplantation. During D‐HOPE, MD glucose and lactate levels increased (ANOVA = 9.88 [*p* = 0.01] and 3.71 [*p* = 0.08]). Their 2nd‐hour levels were higher in EAD group and positively correlated with L‐GrAFT score. 2nd‐hour MD glucose and lactate were also positively correlated with cold ischemia time, macrovesicular steatosis, weight gain during D‐HOPE, and perfusate FMN. These correlations were not apparent when perfusate levels were considered. In contrast, MD FMN levels invariably dropped steeply after D‐HOPE start, whereas perfusate FMN was higher in dysfunctioning grafts.

**Conclusion:**

MD glucose and lactate during D‐HOPE are markers of hepatocellular injury and could represent additional elements of the viability assessment.

## Graphical abstract

In this study, Patrono et al. explore release of metabolites in extracellular space during hypothermic oxygenated machine perfusion by the mean of microdialysis. Levels of glucose and lactate in the microdialysate correlate with ischemia‐reperfusion injury and could represent additional markers of liver viability during hypothermic perfusion.

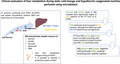

## 1 INTRODUCTION

Hypothermic oxygenated machine perfusion (HOPE) is gaining increasing interest as a tool to reduce ischemia‐reperfusion injury and to improve outcomes of liver transplantation (LT). In clinical studies, HOPE use has been associated with improved outcomes of grafts from donors after circulatory death (DCD),^1^, ^2^, ^3^ extended‐criteria donors after brain death (DBD)^4^, ^5^, and steatotic grafts.^6^ The protective mechanism of HOPE appears to be manifold, being related to adenosine‐triphosphate replenishment, immunomodulation,^7^ better preservation of endothelial cells glycocalyx, peri‐biliary vascular plexus, and peribiliary glands,^8^, ^9^ and, more importantly, modulation of mitochondrial respiration. As shown in 2013 by Schlegel et al.,^10^ HOPE progressively decreases the rate of mitochondrial respiration and determines an oxidized state in mitochondria, limiting the production of reactive oxygen species by reverse electron transfer upon organ reperfusion,^11^, ^12^ thus resulting in diminished mitochondrial, nuclear, hepatocyte, and sinusoidal injury.

Little is known, however, about other aspects of liver metabolism during HOPE, especially concerning the transition between the phase of static cold storage (SCS) and HOPE.

Microdialysis (MD) is a technique by which interstitial fluid (microdialysate) can be sampled from a variety of tissues to measure the concentration of metabolites such as glucose, lactate, pyruvate, and glutamate, which diffuse into the extracellular space. MD has been extensively used in a variety of settings, especially for bedside sampling of cerebral interstitial fluid in critically ill patients.^13^, ^14^, ^15^, ^16^, ^17^ In LT, MD has been used to assess liver metabolism during the phases of liver retrieval, cold preservation and after graft implantation,^18^, ^19^, ^20^, ^21^, ^22^ and explored as a tool for early detection of ischemic complications and acute rejection.^23^, ^24^, ^25^, ^26^, ^27^, ^28^, ^29^ As applied to machine perfusion, MD has the potential of allowing monitoring of liver metabolism throughout SCS and HOPE, overcoming one limitation of perfusate analysis, which cannot assess metabolism during SCS. In a study from our group, MD was used to assess the metabolism of lungs perfused ex‐vivo and emerged as a potential tool to discriminate lung function after transplantation.^30^ MD has been occasionally used in the setting of kidney machine perfusion^31^, ^32^, ^33^ but, to the best of our knowledge, its use in HOPE‐treated livers has not been reported.

The aim of this study was to assess the time course of liver metabolism biomarkers during SCS and HOPE using MD and to explore the potential role of MD for liver graft viability assessment in LT.

## 2 PATIENTS AND METHODS

### 2.1 Study design

This was a prospective, open‐label observational pilot study on 10 consecutive grafts treated with dual‐HOPE (i.e., double cannulation of both portal vein and hepatic artery—D‐HOPE) in the period October 2019–January 2020 at our Institution. The study was approved by the local ethics committee (resolution nr. 739 of June 10, 2019). Patients signed a consent form for receiving an organ treated with machine perfusion and for participating in the study. All study procedures complied with the Declaration of Helsinki and the Declaration of Istanbul (https://www.wma.net). Recipients of DBD grafts included in this study were also included in a recently published study on the value of perfusate analysis during D‐HOPE in predicting outcome after LT.^34^

Our machine perfusion and LT protocols have been previously described.^4^, ^34^, ^35^, ^36^ At our Institution, the use of D‐HOPE is systematic for grafts from DCD donors, whereas it is evaluated on a case‐by‐case basis for grafts from DBD donors, mainly based on donor age and steatosis. In this study, end‐ischemic D‐HOPE using LiverAssist^®^ (XVIVO, Groningen, The Netherlands) primed with 3 L of Belzer MP^®^ fluid (Bridge to Life Europe Ltd. London, UK) was applied for a minimum of 90 min during recipient hepatectomy. The liver graft was weighed before and after machine perfusion to detect swelling during machine perfusion.

**FIGURE 1:**
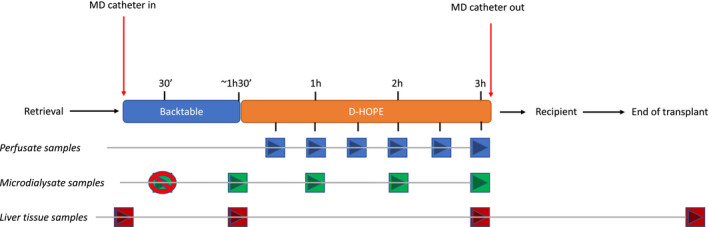
Synopsis of study design [Color figure can be viewed at wileyonlinelibrary.com]

MD was used to sample extracellular fluid during back table preparation and subsequent machine perfusion. Study design and timing of sample collection are summarized in Figure 1. Briefly, the liver was perfused with Celsior^®^ (IGL, Lisseu, France) at retrieval and transported in SCS at our center. The liver was unpacked upon arrival and, before the start of back table preparation, a 61 hepatic microdialysis catheter^®^ with membrane length = 30 mm and membrane cutoff ~20 kDa (M Dialysis AB, Stockholm, Sweden) was inserted at a ~4 cm depth by the mean of a splitable introducer into liver segment 6 and secured by a 5/0 Prolene suture. The MD catheter was connected to a 107 Microdialysis pump^®^ charged with normal saline with a flow set at 2 μl/min (after an initial 5‐min flush at 15 μl/min). By this setting, the concentration of metabolites in microdialysate represents roughly 40% of extracellular fluid concentration.^37^ The first MD vial was connected to the MD catheter after the first drop of microdialysate appeared at the tip of the connection needle and discarded after 30 min to allow fluid equilibration, as recommended.^13^, ^38^, ^39^ The second vial, which was the first to be analyzed, was collected at the end of backtable preparation, at least one hour after having been positioned, and was therefore representative of liver metabolism during the last part of SCS. Subsequently, the liver was connected to the perfusion device with the MD catheter in place and MD vials were changed hourly during D‐HOPE. Thus, at least 3 MD samples were available for every single procedure: the first representing the terminal phase of SCS, and the remaining two samples the D‐HOPE phase. When D‐HOPE time was <120 min, the last MD sample was collected at the end of machine perfusion, thus representing extracellular fluid concentration during the 2nd hour of D‐HOPE.

During D‐HOPE, perfusate samples were collected every 30 min from the sampling port on the hepatic artery circuit and stored in cryotubes.^34^ Both MD and perfusate samples were snap frozen and subsequently analyzed using CMA 600 Microdialysis Analyzer. The concentration of glucose, lactate, pyruvate, and glutamate was measured, and lactate/pyruvate ratio was calculated. Perfusate parameters were normalized to liver weight. Perfusate and microdialysate flavin mononucleotide (FMN) level was measured with Synergy HTX microplate reader (BioTek Instruments, Winooski, VT, USA) using an excitation wavelength of 460/40 nm and recording the fluorescence with wavelength of 528/20 nm with 100% gain, as previously described.^40^, ^41^ FMN levels were expressed as relative fluorescing units (RFU) and, given the methodology used for their measurement, are presented both as non‐adjusted and adjusted values. The MD catheter was removed at the end of machine perfusion. Liver biopsies were collected at the beginning of backtable preparation, before and after D‐HOPE, and at the end of the transplant. Biopsies were immediately immersed in RNA Later solution (Invitrogen, Thermofisher) and freezed at −20°C for subsequent determination of interleukin‐6 (IL‐6), interleukin‐8 (IL‐8), tumor necrosis factor α (TNFα), toll‐like receptor‐4 (TLR‐4), intercellular adhesion molecule 1 (ICAM1) and C‐X‐C Motif Chemokine Ligand 12 (CXCL12) expression (Table S1). Succinate level was measured on hepatic biopsies using the Succinate Colorimetric Assay Kit (K649, Biovision Incorporated, Milpitas, CA, USA) (Supporting Information). Histological ischemia‐reperfusion injury and steatosis were determined on liver biopsies obtained at the end of transplant. Tissue samples were fixed and processed to obtain 5‐µm‐thick sections. Hematoxylin and eosin, periodic acid‐Schiff, and periodic acid‐Schiff‐diastase staining were performed to evaluate steatosis, necrosis, as well as glycogen content and distribution.

D‐HOPE and MD were not used for graft evaluation and all livers in this series were transplanted. A t‐tube was routinely positioned during transplant operation and removed at 3 months after a control cholangiogram.

Collected variables included indication for LT, recipient, and donor features, as well as times and technical details about retrieval, initial SCS, D‐HOPE, and transplant operation. Levels and trend of microdialysate and perfusate metabolites were described in the whole cohort and analyzed with regards to their correlation with outcome measures, as outlined below.

### 2.2 Outcome measures

Primary outcome measures were early allograft dysfunction (EAD)^42^ and L‐GrAFT score.^43^ Secondary outcome measures were duration of hospital stay, onset and grade of acute kidney injury (AKI),^44^ and comprehensive complication index^45^ (CCI) calculated at hospital discharge and at 6‐month follow‐up. Graft survival and occurrence of biliary complications were also evaluated. Minimum follow‐up was 6 months.

### 2.3 Statistical analysis

Variables were expressed as number (%) or median (interquartile range) and compared using standard parametric and non‐parametric tests. All perfusate values were normalized to liver weight. For repeated measures ANOVA, normal distribution of variables was verified by Shapiro–Wilk test and by visual inspection using quantile‐quantile plots. *p*‐values of pairwise *t*‐tests between different timepoints were adjusted using the Bonferroni multiple testing correction method. Correlation between variables was evaluated using Pearson correlation coefficient. Optimal cutoff points in ROC analyses were calculated using Youden method. Considering the exploratory nature of this study, a convenience sample size = 10 was chose based on the number of D‐HOPE procedures per year at our Institution. All analyses were performed using R version 3.6.3 (R Foundation for Statistical Computing, Vienna, Austria. https://www.R‐project.org/).

## 3 RESULTS

### 3.1 D‐HOPE procedure and clinical outcome

Recipient and donor features are summarized in Table 1. Machine perfused grafts were preferentially allocated to low MELD (12.1 [9.1, 14.5]) patients, of whom 7 (70%) had hepatocellular carcinoma. The indication for D‐HOPE was based on advanced donor age (cases 6, 7, 8, and 10), high BMI and steatotic graft appearance (cases 3, 4, 5, and 9), advanced donor age in association with graft steatosis (case 1) and donation after circulatory death (case 2). Table 2 summarizes times and graft weight before and after machine perfusion. Cold ischemia and D‐HOPE time were 344 (295, 367) and 116 (103, 143) min. In 3 grafts weight increased ≥5% during D‐HOPE. Total preservation time never exceeded 10 h 15 min.

**TABLE 1:** Baseline characteristics of recipients and donors

Recipient features							Donor features				
*n*	S	Age (years)	BMI	Indication	HCC	MELD	Age (years)	Type	BMI	M (%)	μ (%)
1	M	68	27	ETOH+NASH	No	15	75	DBD	45	20	70
2	M	60	27	PBC	Yes	11	60	DCD.3	26	1	5
3^a^	M	62	33	ETOH+NASH	yEs	8	48	DBD	37	25	30
4^b^	M	53	33	HCV+NASH	Yes	20	63	DBD	29	40	30
5	M	66	22	ETOH	No	13	58	DBD	41	5	35
6^a^	F	64	21	ETOH	No	13	83	DBD	23	10	60
7	F	63	26	HBV+NASH	Yes	9	83	DBD	24	3	40
8	F	61	18	HBV+HDV	Yes	18	80	DBD	27	10	70
9	M	67	27	NASH	Yes	9	53	DBD	34	1	2
10	F	66	28	NASH	Yes	9	83	DBD	20	0	5
Median		63	23			12	69		28	7	32
25th %		61	27			9	59		24	1	11
75th %		66	28			14	82		36	17	55

Abbreviations: BMI, body mass index; Crea, creatinine; DBD, donation after brain death; DCD, Maastricht category 3 donation after circulatory death; ETOH, alcoholic cirrhosis; GGT, gamma glutamyl transferase; HBV, hepatitis B virus; HCC, hepatocellular carcinoma; HCV, hepatitis C virus; HDV, hepatitis D virus; M, macrovesicular steatosis; Na, sodium; NASH, non‐alcoholic steatohepatitis; PBC, primary biliary cholangitis; S, sex; μ, microvesicular steatosis.

^a^ Developed early allograft dysfunction.

^b^ Developed graft failure.

**TABLE 2:** Operational characteristics of liver grafts

*n*	Retr. (min)	CIT^a^ (min)	BT (min)	D‐HOPE (min)	Weight pre (g)	Weight post (g)	Delta weight (%)	rWIT^b^ (min)	Tot (min)
1	47	341	95	106	1550	1480	−4	30	491
2	37	360	94	147	1370	1370	0	23	550
3	57	369	130	103	2290	2410	+5	25	508
4	80	443	120	131	2400	2800	+17	37	614
5	67	407	135	103	1980	2090	+6	21	530
6	48	291	72	151	1500	1480	−1	24	497
7	41	296	115	125	1180	1120	−5	21	453
8	35	295	105	190	1000	1010	+1	24	524
9	43	348	170	91	1750	1760	+1	21	474
10	50	268	110	101	940	960	+2	22	404
Median	47	344	112	116	1525	1480	0.8	23	502
25th %	41	295	97	103	1227	1182	−1.0	21	478
75th %	55	367	127	143	1922	2007	4.4	25	528

Abbreviations: BT, back table preparation; CIT, cold ischemia time; D‐HOPE, dual hypothermic oxygenated machine perfusion; Retr, donor hepatectomy time; rWIT, recipient warm ischemia time; Tot, total preservation time.

^a^ Cold ischemia time from cold perfusion in the donor to D‐HOPE start.

^b^ Time from start of vascular anastomoses to graft reperfusion into recipient. The liver was weighed before and after machine perfusion and the weight variation was expressed as a percentage of the initial weight.

Median duration of MD monitoring, from insertion of the first microvial to removal of the last one, was 228 (210, 245) min. No major adverse events occurred and occasional mild bleeding from MD catheter entry site was easily controlled by diathermy. No patient developed catheter‐related bleeding or intra‐or extra‐hepatic hematoma, as assessed by ultrasound scan performed after LT.

Study variables and outcome measures are summarized in Table 3. Three patients developed EAD after LT and one later developed delayed non‐function and required retransplantation. All three patients had a transaminase peak ≥2000 IU, whereas only the patient who subsequently suffered from graft failure had bilirubin level ≥10 mg/dl on day 7th after LT. Patients who developed EAD had higher transaminase peak, as well as higher day 7th bilirubin, INR, and alkaline phosphatases levels (Table 3). L‐GrAFT score was higher in the EAD group, with a difference approaching statistical significance (estimated risk of graft loss 22.1% vs. 7.9%, *p* = 0.09).

**TABLE 3:** Study variables in the whole series and according to the onset of early allograft dysfunction

	Overall	EAD	No EAD	*p*
	10	3	7	
Recipient age (years)	63.5 [61.0, 66.3]	62.1 [57.7, 63.0]	65.8 [61.8, 66.6]	0.21
Recipient BMI	26.8 [23.1, 28.1]	33.1 [27.1, 33.1]	26.7 [24.1, 27.1]	0.30
Donor age (years)	69.1 [58.6, 82.2]	63.5 [55.9, 73.4]	74.8 [59.1, 81.5]	0.73
Donor BMI	28.0 [24.4, 36.1]	29.3 [26.2, 33.0]	26.8 [25.0, 37.6]	0.91
Donor ICU stay (days)	4.0 [3.2, 5.5]	4.0 [3.0, 5.0]	4.0 [3.5, 5.5]	0.72
Donor GGT (IU/L)	25 [18, 46]	32.00 [25, 41]	23 [15, 93]	0.65
Macrosteatosis (%)	7.5 [1.5, 17.5]	25.0 [17.5, 32.5]	3.0 [1.0, 7.5]	0.04
Microsteatosis (%)	32.5 [11.2, 55.0]	30.0 [30.0, 45.0]	35.0 [5.0, 55.0]	0.91
Donor hepatectomy (min)	47 [41, 55]	57 [52, 68]	43 [39, 48]	0.09
Cold ischemia time (min)	344 [29, 366]	369 [330, 406]	341 [295, 354]	0.42
Backtable time (min)	112 [97, 127]	120 [96, 125]	110 [100, 125]	0.91
D‐HOPE (min)	113 [103, 123]	120 [111, 120]	106 [98, 136]	1.00
Graft weight pre (g)	1525 [1227, 1922]	2,290 [1895, 2345]	1,370 [1090, 1650]	0.09
Graft weight post (g)	1,480 [1182, 2007]	2,410 [1945, 2605]	1,370 [1065, 1620]	0.07
Delta weight (%)	0.80 [−0.98, 4.43]	5.20 [1.95, 10.95]	0.60 [−2.25, 1.55]	0.30
Total preservation (min)	502 [478, 528]	508 [502, 561]	491 [463, 527]	0.30
Surgery time (min)	365 [287, 455]	395 [354, 455]	335 [278, 448]	0.42
PRBC units	4.0 [4.0, 7.5]	4.0 [4.0, 4.0]	6.0 [2.0, 10.5]	0.48
End lactate (mmol/L)	2.15 [1.48, 2.30]	2.10 [1.60, 2.15]	2.30 [1.65, 2.30]	0.42
Induction (basiliximab)	8 (80.0%)	3 (100.0%)	5 (71.4%)	0.86
Day of IS start				0.24
0	3 (30.0%)	1 (33.3%)	2 (28.6%)	
1	6 (60.0%)	1 (33.3%)	5 (71.4%)	
4	1 (10.0%)	1 (33.3%)	0 (0.0%)	
AST peak (IU/L)	1,020 [676, 1850]	3,375 [2695, 7725]	721 [616, 1020]	0.02
ALT peak (IU/L)	837 [463, 1003]	1,000 [937, 1048]	510 [419, 902]	0.21
Bilirubin day 7th (mg/dl)	2.0 [1.4, 4.8]	5.5 [3.1, 9.0]	2.0 [1.5, 2.4]	0.57
INR day 7th	1.2 [1.2, 1.3]	1.3 [1.3, 1.4]	1.2 [1.2, 1.3]	0.64
ALP day 7th (IU/L)	192.0 [143.8, 231.5]	237.0 [169.0, 340.5]	170.0 [149.5, 214.5]	0.49
L‐GrAFT (risk %)	8.7 [7.7, 19.2]	22.1 [15.6, 51.3]	7.9 [6.3, 9.4]	0.09
AKI stage				0.28
No AKI	2 (20.0%)	1 (33.3%)	1 (14.3%)	
1	5 (50.0%)	1 (33.3%)	4 (57.1%)	
2	2 (20.0%)	0 (0.0%)	2 (28.6%)	
3	1 (10.0%)	1 (33.3%)	0 (0.0%)	
Clavien‐Dindo ≥3 complications	2 (20.0%)	2 (66.7%)	0 (0.0%)	0.12
Hospital CCI	21.7 [11.7, 27.8]	33.5 [16.7, 51.5]	20.9 [14.8, 22.6]	0.42
6‐month CCI	30.6 [22.6, 38.7]	47.4 [40.5, 73.7]	22.6 [21.7, 31.9]	0.05

Note

Data are presented as median [IQR] or number (%), as appropriate.

Abbreviations: AKI, acute kidney injury; ALP, alkaline phosphatase; ALT, alanine aminotransferase; AST, aspartate aminotransferase; BMI, body mass index; CCI, comprehensive complication index; D‐HOPE, dual hypothermic oxygenated machine perfusion; EAD, early allograft dysfunction; GGT, gamma glutamyl transferase; ICU, intensive care unit; INR, international normalized ratio; IS, immunosuppression; L‐GrAFT. Liver graft assessment following transplantation; PVT, portal vein thrombosis; TIPS, transjugular intrahepatic portosystemic shunt.

Concerning baseline variables, the only significant difference between EAD and non‐EAD cases was a higher percentage of macrovesicular steatosis (25% vs. 3%, *p* = 0.04) in EAD group. Degree of macrovesicular steatosis was 25% and 10% in patients who recovered from EAD, whereas it was 40% in the patient who developed graft failure.

With regards to clinical outcome, patients in the EAD group had in the general poorer outcome and suffered from a higher rate of surgical complication postoperatively and at 6‐month follow‐up, as demonstrated by a higher CCI (47.4 vs. 22.6, *p* = 0.05). Only patient 4 developed Clavien‐Dindo ≥3b complications, represented by coagulopathy‐related bleeding requiring relaparotomy and temporary packing on postoperative day 1, followed by renal failure requiring renal replacement therapy and graft failure leading to retransplantation on postoperative day 31st.

Four (40%) patients developed biliary complications (anastomotic, *n* = 3; ischemic‐type, *n* = 1). Patients 3, 8, and 10 presented at 3‐month cholangiogram with sludge and anastomotic stricture, which were successfully managed endoscopically. Patient 6 developed an ischemic‐type stricture of the biliary confluence, which was successfully managed by percutaneous balloon bilioplasty and no evidence of recurrence thereafter.

Median follow‐up was 10.9 (9.8, 11.6) months. Patient and graft survival were 90%. Patient 4, after making a good recovery after re‐LT, died 6 months after LT due to complications of HHV8 infection.

### 3.2 Microdialysate and perfusate metabolites

**FIGURE 2:**
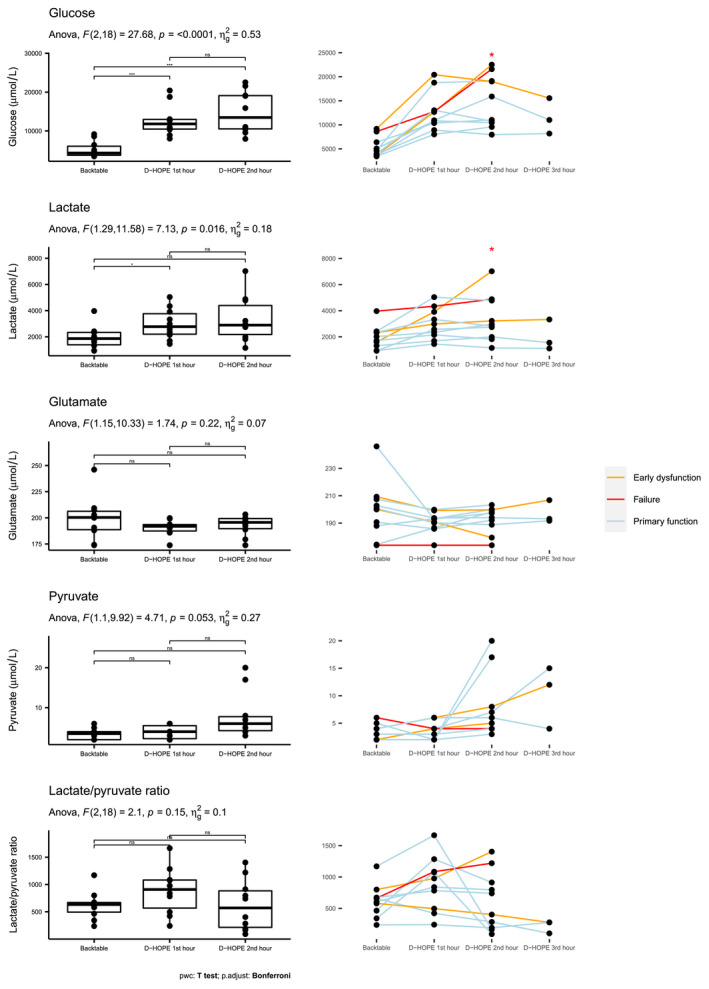
Glucose, lactate, glutamate, pyruvate levels and lactate/pyruvate ratio in microdialysate during backtable preparation and subsequent machine perfusion. In the left column, levels across different timepoints are compared using ANOVA for repeated measures. Degrees of freedom, *F*‐statistic (*F*), *p*‐value, and generalized effect size squared (*η* ^2^) are provided for each biomarker. Levels of significance of pairwise *t*‐test across different time points is indicated as non‐significant (ns), <0.01 (**) or <0.001 (***). *Y* axis scale changes across different plots to improve data visualization. As only 3 grafts had a D‐HOPE time exceeding 2 h, the 3‐h timepoint is not visualized. In the right column, line plots depicting the trend of study metabolites in each patient are provided. Line colors identify patients who had primary graft function (light blue), early allograft dysfunction (orange), or required retransplantation (red). 2nd hour samples were collected 2 h after the beginning of D‐HOPE or at the end of machine perfusion when D‐HOPE time was <120 min. Asterisks indicate that glucose and lactate levels during the 2nd hour of D‐HOPE were significantly higher in patients developing early allograft dysfunction. pwc, pairwise comparison [Color figure can be viewed at wileyonlinelibrary.com]

Figure 2 depicts values of MD metabolites during SCS and D‐HOPE. Only 3 patients had D‐HOPE lasting >2 h and had samples representative of this time frame. We observed a significant rise of MD glucose level upon initiation of D‐HOPE, which increased from 49 (42, 68) mg/dl to 133 (118, 146) and 152 (119, 216) mg/dl at 1st and 2nd hour of D‐HOPE, respectively (*p* = 0.01). A similar trend was observed for lactate, which increased from 1. 9 (1.4, 2.3) mmol/L to 2.8 (2.2, 3.8) mmol/L at 1st hour and 2.9 (2.2, 4.4) mmol/L at 2nd hour (*p* = 0.08). Levels of glutamate in MD fluid persisted high throughout the procedure (200 [189, 206], 192 [187, 194], and 196 [190, 199] μmol/L during SCS, D‐HOPE 1st and 2nd hour, respectively) and were unaffected by D‐HOPE, whereas pyruvate levels were persistently low (4 [2, 4], 4 [2, 6], and 6 [4, 8] μmol/L at all timepoints, respectively). As an effect of low pyruvate levels, lactate/pyruvate ratio trend closely mimicked the lactate trend.

**FIGURE 3:**
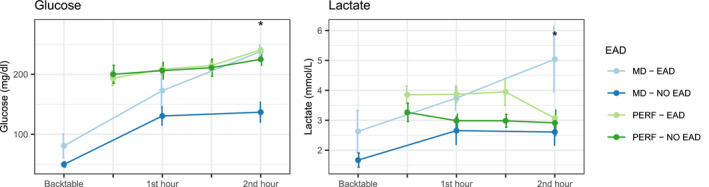
Line plots depicting trend of glucose and lactate in perfusate (green and light green) and microdialysate (blue and light blue), according to subsequent development of early allograft dysfunction. In cases in which D‐HOPE time was <120 min, 2nd hour samples were collected at the end of machine perfusion. Values are represented as mean ± standard error (vertical error bars) [Color figure can be viewed at wileyonlinelibrary.com]

Kinetics of MD lactate and glucose was different in grafts that developed EAD (Figure 2). In particular, levels of glucose and lactate were significantly higher during 2nd hour of D‐HOPE (244 vs. 121 mg/dl, *p* = 0.03 and 4.9 vs. 2.7 mmol/L, *p* = 0.03) (Table S2). In contrast with glucose and lactate levels on perfusate, levels of MD metabolites clearly diverged, being significantly higher in dysfunctioning grafts at two hours of machine perfusion (Figure 3) (Tables S2 and S3). For 2nd‐hour MD glucose, the area under the receiver operating characteristic curve evaluating its association with EAD was 0.952. With a cutoff value of 215 mg/dl, 2nd hour MD glucose had 100% sensitivity, 86% specificity, 75% positive predictive value, and 100% negative predictive value for the development of EAD.

**FIGURE 4:**
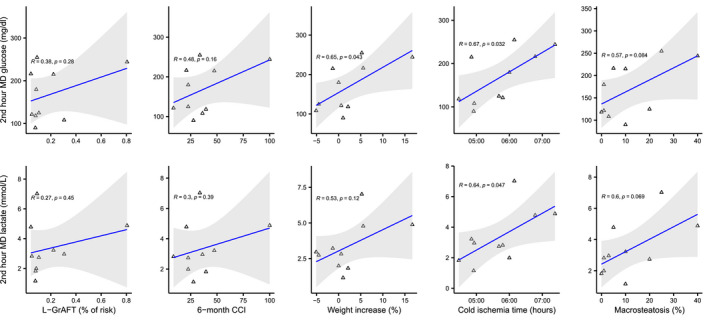
Correlation of 2nd hour MD metabolites with L‐GrAFT score, 6‐month CCI, and graft characteristics [Color figure can be viewed at wileyonlinelibrary.com]

2nd‐hour MD glucose and lactate level were positively correlated with L‐GrAFT score, 6‐month CCI, graft weight variation during D‐HOPE, cold ischemia time, and macrosteatosis (Figure 4).

Levels of MD metabolites were not associated with the development of biliary complications. However, both surviving grafts that initially developed EAD subsequently developed biliary complications, including one case of ischemic cholangiopathy.

### 3.3 Microdialysate and perfusate FMN

**FIGURE 5:**
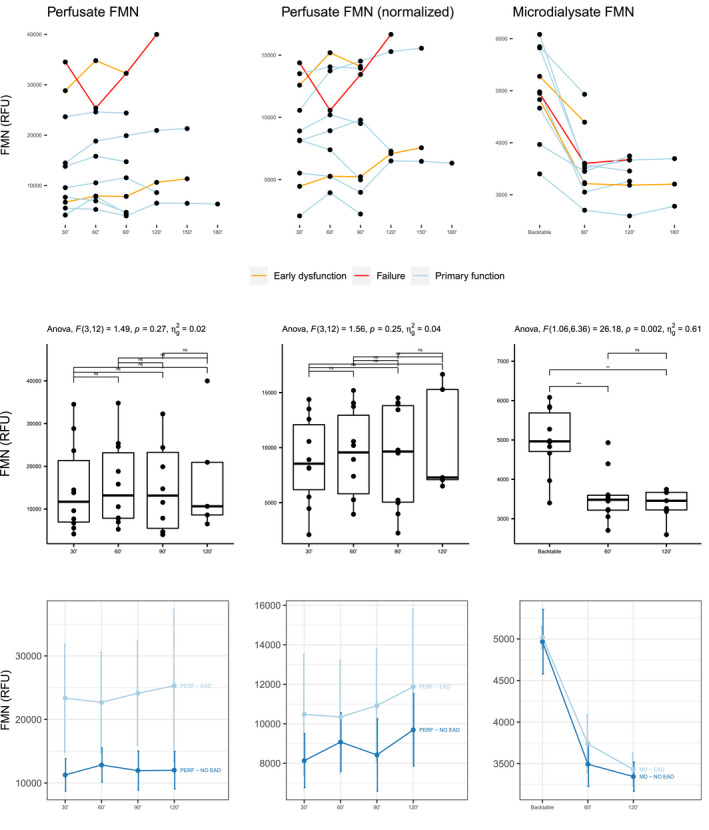
Non‐adjusted (left column) and adjusted (middle column) flavin mononucleotide level in perfusate and in microdialysate (right column). Individual trends for each patient are presented in the first row, with colors differentiating cases according to early graft function. Levels across different timepoints are compared with ANOVA for repeated measures in the second row. In the third row, levels (mean ± standard error) are presented according to the development of early allograft dysfunction [Color figure can be viewed at wileyonlinelibrary.com]

**FIGURE 6:**
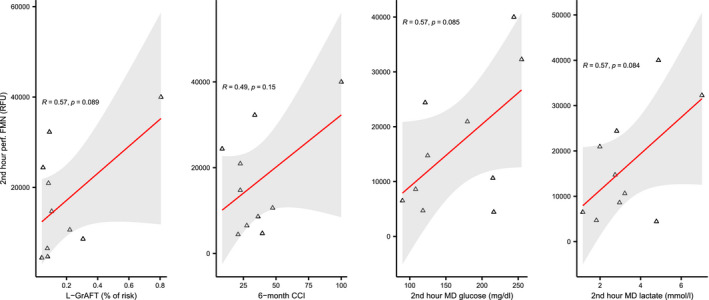
Correlation of 2nd hour perfusate FMN with L‐GrAFT score, 6‐month CCI, and 2nd hour MD metabolites [Color figure can be viewed at wileyonlinelibrary.com]

Figure 5 depicts FMN levels in perfusate and microdialysate. As opposed to glucose and lactate, FMN levels in microdialysate significantly dropped after initiation of D‐HOPE, independently of graft function. In contrast, adjusted and non‐adjusted perfusate FMN levels were higher in grafts who developed early dysfunction, although this difference did not achieve statistical significance (Table S4). The only graft that developed delayed non‐function and required re‐LT was characterized by the highest perfusate FMN levels, which progressively increased throughout D‐HOPE. Perfusate FMN levels were positively correlated with L‐GrAFT score, 2nd‐hour MD glucose and lactate, and with clinical outcome measures (Figure 6).

### 3.4 Histology and expression of inflammatory cytokines

**FIGURE 7:**
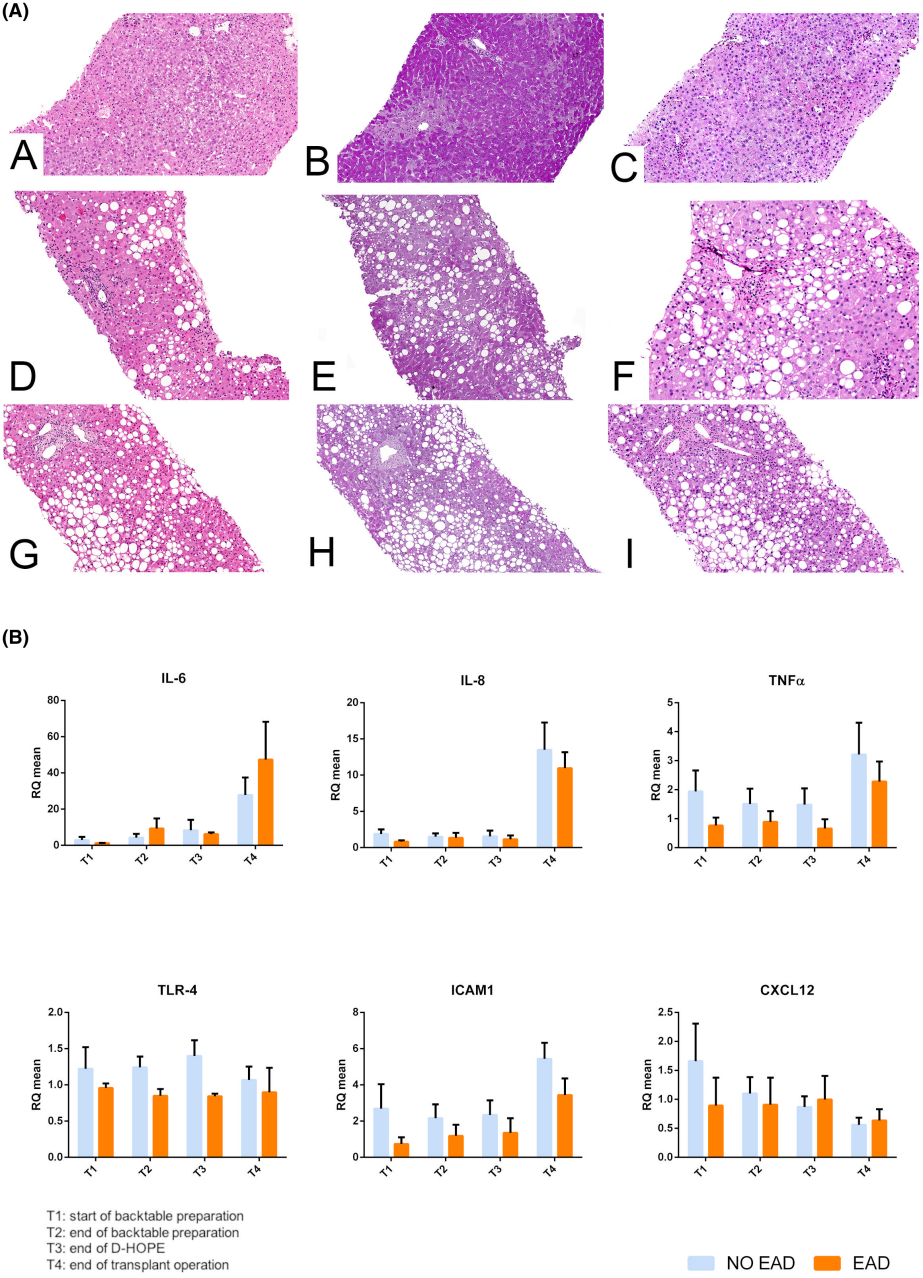
Panel A. Representative histological images of liver grafts at the end of transplant (100× original magnification). Non‐EAD case (case 10; images A–C) showed mild signs of steatosis and reperfusion injury (A). PAS staining (B) enhanced cytoplasmatic hepatocytes’ glycogen deposits, confirmed with PAS‐D staining (C). Glycogen was diffusely distributed with a zone‐1‐to‐zone‐3 gradient pattern. EAD case (case 3; images D–F) was characterized by mild steatosis and focal parcellar necrosis (D). Glycogen was present in few periportal hepatocytes (E,F). Graft failure case (case 4; images G–I) showed severe steatosis (G) and minimal signs of glycogen deposits (H,I). Microscope liver histologic slides were scanned with the NanoZoomer S210 Digital slide scanner (Hamamatsu Photonics K.K.) using an objective lens with a numerical aperture of 0.75. Slides were focused at 400× original magnification (scanning resolution: 0.23 μm/pixel), and images were acquired with the NDP.scan image acquisition software (Hamamatsu Photonics K.K.). Then, contrast and brightness corrections were performed to the whole image and data exported with the NDP.view2 viewing software (Hamamatsu Photonics K.K.). Panel B. Cytokines levels at the start of backtable preparation (T1), before (T2) and after (T3) machine perfusion, and at the end of transplant operation (T4). No significant differences were observed between EAD and non‐EAD patients at any timepoint [Color figure can be viewed at wileyonlinelibrary.com]

Histological injury (Figure 7—Panel A) correlated with EAD onset and was more severe in the graft that required retransplantation. Interestingly, reduced glycogen content was observed in grafts that subsequently developed EAD. Upon reperfusion, a significant increase of inflammatory cytokines (IL‐6, IL‐8, and TNFα) and adhesion molecules (ICAM1) expression was observed, with no significant differences according to EAD development (Figure 7—Panel B). There was no correlation between MD and perfusate parameters and the expression of inflammatory cytokines or adhesion molecules. Succinate tissue content exhibited a downward trend from cold preservation to reperfusion into recipient, with no significant differences between study groups (Figure S1).

## 4 DISCUSSION

In the setting of clinical LT, the use of MD has been explored mainly as a tool for monitoring and early detection of ischemic complications and acute rejection.^23^, ^24^, ^25^, ^26^, ^27^, ^28^, ^29^ However, one fascinating feature of this technology is the possibility of evaluating liver metabolism during different phases of organ retrieval, static cold storage, implantation, and early postoperative course.^18^, ^19^, ^21^, ^22^ In particular, the studies by Nowak et al.^19^ and Silva et al.^21^ have clearly shown that both glucose and lactate slowly build up during SCS, whereas their levels increase steeply during implantation and organ reperfusion, to return progressively to baseline levels within 3 h following reperfusion. Perera et al.^46^ have shown that end‐ischemic interstitial lactate level and lactate/pyruvate ratio are higher in grafts from DCD donors, this finding being associated with more severe glycogen depletion. Similarly, glutamate also appears to accumulate during SCS, reaching end‐ischemic levels of approximately 300 μmol/L, which progressively normalize in the hours following graft reperfusion.^20^ In contrast, pyruvate quickly becomes undetectable during SCS (reflecting lack of metabolic activity during this phase), rises above pre‐SCS values upon reperfusion (suggesting a phase of hypermetabolism) to return to baseline in the following hours.^19^

The release of glucose in the extracellular space represents a hepatic‐specific response to ischemia and it has been interpreted as a result of glycogen breakdown.^19^ In other tissues, ischemic events are associated with very low interstitial glucose. Interestingly, the rate of glucose and lactate accumulation increases during implantation^19^ and back table preparation,^21^ suggesting a potential role for temperature during these phases. Previous studies have suggested a prognostic value of MD metabolites, as patients developing initial poor function had higher MD lactate level during back table preparation and delayed clearance after graft reperfusion.^21^, ^46^

In this study, we sought to use MD to deepen our understanding of liver metabolism during SCS and D‐HOPE. Our main finding is that glucose and lactate are released in the extracellular space upon initiation of D‐HOPE and their levels correlate with other known predictors of graft function (cold ischemia time and macrosteatosis), FMN perfusate level, graft dysfunction, and worse clinical outcome.

In our study, D‐HOPE did not alter pyruvate and glutamate levels, as compared to the expected kinetics during SCS. In particular, pyruvate levels were almost undetectable and glutamate levels were constantly high, in keeping with findings from previous studies.^20^ The lower end‐ischemic lactate (~1900 μmol/L) and glutamate (~200 μmol/L) concentrations observed in our study (~200 μmol/L), as compared to those observed in previous studies by Silva et al.^20^, ^21^ can be explained by the relatively short ischemia time before D‐HOPE (5 h 44 min) in our series. Interestingly, the only graft that failed in our series had constantly very low levels of MD glutamate during back table and D‐HOPE.

Why D‐HOPE was associated with a significant increase of glucose and lactate into microdialysate is open to several interpretations. Temperature may have played a role, as the device employed for machine perfusion operates at ~10°C. During rewarming, it is possible that increased metabolism enhanced glycogenolysis, which in turn resulted in increased glucose release. The composition of the perfusion fluid used for D‐HOPE, containing 180 mg/ml of dextrose, may also have influenced MD glucose level. However, it should be noted that glucose levels in microdialysate were lower than those in perfusate and their kinetics was different (Figure 3). In addition, perfusion fluid composition was unlikely to affect lactate levels and progressive accumulation of lactate in the renal cortex has also been observed in a study using MD in a model of hypothermic perfusion of porcine kidneys.^33^

Whatever the mechanism, glucose and lactate released in the extracellular space during D‐HOPE can be interpreted as markers of graft injury. The rise of MD glucose and lactate during the first 2 h of D‐HOPE was more important in grafts that developed early dysfunction, whereas it was mild or absent in those exhibiting primary function (Figure 3). However, in the few cases that had a 3‐h MD sample available, glucose and lactate levels started to decrease also in EAD group (Table S2). Consequently, it seems that restoration of aerobic metabolism during D‐HOPE could take longer in more severely damaged grafts.

Importantly, there was a significant discrepancy between perfusate and MD kinetics of glucose and lactate (Figure 3). Perfusate analysis, as a measure of larger and global compartment, did not seem to entirely capture metabolic changes happening at a cellular level, a finding that was more evident for glucose and which is in keeping with our previous study on ex‐vivo lung perfusion.^30^ Perfusate glucose level increased during D‐HOPE independently of graft function, whereas MD levels of glucose were higher in EAD patients, with a significant difference during the 2nd hour of machine perfusion. As lactate is produced during anaerobic glycolysis and glucose is released during ischemia due to hepatocytes glycogenolysis, we might hypothesize that the grafts that developed EAD did not fully recover after SCS and continued to experience a certain degree of hypoxia during machine perfusion, as demonstrated by their metabolic profile. In line with this observation, glycogen content was reduced in EAD livers, likely due to glycogen depletion during cold preservation, an observation which is in keeping with findings from Perera et al.^46^

The next logical step was comparing these findings with perfusate and MD levels of FMN, a marker of mitochondrial injury that recently emerged as a promising tool for graft viability assessment.^40^, ^41^ As opposed to glucose and lactate, D‐HOPE start determined a steep decrease of MD FMN levels, suggesting its rapid washout from the extracellular space and confirming protection of mitochondria during D‐HOPE. In contrast, FMN appeared to accumulate in perfusate, with an incremental trend in grafts who developed EAD and with the highest levels observed in the only failing graft of this series (Figure 5). This finding, which is in keeping with those from Mueller et al.,^40^ also suggests a mechanistic interpretation to the association between microdialysate metabolites and graft dysfunction. Taken as a whole, our data seem to point to the same direction: mitochondrial injury sustained during SCS, which is proportional to cold ischemia time and macrosteatosis, is reflected by the accumulation of FMN in perfusate and release of glucose and lactate in the extracellular space during D‐HOPE, highlighting their potential as viability markers. Noteworthy, these metabolites can be measured point‐of‐care using MD equipment during D‐HOPE.

This study has numerous implications. First, it confirms that precious information on graft function and injury can be gathered also during hypothermic perfusion, challenging the concept that normothermic perfusion is a prerequisite for graft viability assessment.^47^, ^48^ Second, while perfusate analysis appears handier, this study and previous experiences^23^, ^24^, ^27^, ^28^ suggest that MD is feasible without major modifications of routine practice. In our opinion, microdialysate and perfusate analysis should be seen as complementary rather than alternative techniques, as metabolites kinetics appears to be different in these two compartments. Real‐time assessment of graft function could drive not only graft acceptance but also influence the duration of machine perfusion, as more severely damaged grafts may require longer perfusion time to recover from ischemic injury.

Third, this pilot study represents a baseline based on which other potential applications of MD during machine perfusion could be explored. In a recent study on perfusate analysis during D‐HOPE,^34^ we identified alanine aminotransferase as the most reliable predictor of EAD. Unfortunately, MD ALT could not be evaluated due to its molecular weight (100 kDa). Further studies on the subject should ideally employ available 100 kDa MD catheters to allow measurement of other molecules, including inflammatory cytokines, as also suggested by a recent study.^49^ Finally, MD could be used to assess liver metabolism and explore new viability criteria during normothermic machine perfusion, a setting mimicking normal physiology and characterized by longer perfusion times.^50^

Limitations of our study include the small sample size of 10 grafts with rather heterogeneous characteristics, the lack of a power analysis, and the choice of EAD as an endpoint, which has been discouraged in machine perfusion trials as it strongly relies on transaminase level after transplant and could be influenced by the washout phenomenon during machine perfusion.^51^

In this study, microdialysis time was limited to less than 4 h by design, allowing complete measurement of MD metabolites at only 3 time points. As previous studies have shown a quick recovery of aerobic metabolism after liver reperfusion^21^, ^46^ and due to concerns about sterility breaches, we did not consider leaving the MD in place during this phase, possibly missing relevant metabolic changes.^31^ Rapid sampling MD^33^ could improve the yield of MD as applied to procedures of limited duration, whereas the use of an MD catheter with higher molecular weight cutoff membrane would allow measuring MD concentration of other potentially important molecules.

In conclusion, this study expands previous knowledge on liver metabolism during D‐HOPE and confirms that liver graft injury and function can be assessed during hypothermic machine perfusion. These preliminary findings require validation in larger studies allowing correlation of MD parameters with clinically relevant endpoints and possibly exploring further applications of MD in machine perfusion.

## CONFLICT OF INTEREST

The authors of this manuscript have no conflicts of interest to disclose.

## AUTHOR CONTRIBUTIONS

Damiano Patrono: concept and design, data collection, analysis, and interpretation, statistics, drafting article; Dorotea Roggio: data analysis and interpretation, drafting article; Anna Teresa Mazzeo: concept and design, data interpretation, funding, approval of article; Giorgia Catalano, Elena Mazza, Giorgia Rizza, Alessandro Gambella, Federica Rigo, Nicola Leone, Vincenzo Elia, Daniele Dondossola, Caterina Lonati: data collection, analysis, and interpretation; article revision; Vito Fanelli: concept and design, article revision; Renato Romagnoli: concept and design, funding, article revision, study supervision.

## Supporting information

Supplementary Material
