# Supplementary material for: Clinical assessment of liver metabolism during hypothermic oxygenated machine perfusion using microdialysis
Source: Artif Organs. 2021 Sep 21;46(2):281–95. doi: 10.1111/aor.14066 (PMC9292750; doi:10.1111/aor.14066)
Supplement: Supplementary file 1 — Supplementary Material [file AOR-46-281-s001.docx]

Supplementary material

**Hepatic biopsies collection**

Wedge hepatic biopsies were collected four times during all the procedure: start of backtable (T1), end of backtable-start of HOPE (T2), end of HOPE (T3), post graft transplant (T4). Each biopsy was immediately immerged in RNA Later solution (Invitrogen, Thermofisher) and freezed at -20°C, then used for subsequent analysis.

**RNA extraction and RT-PCR**

An equal amount of each biopsy (50 mg) was suspended in 500 ul of TRIzol™ solution (Invitrogen, Thermofisher) together with 0.5 mm size zirconium beads, then homogenized by Bullet Blender (Next Advance, Inc), at a speed of 8 rpm for 3 minutes at 4°C. The homogenized tissue was centrifuged at 12,000 g for 15 min at 4°C and the collected supernatant was subjected to RNA extraction according to manufacturer’s protocol. Briefly, 200 μl of chloroform were added to each sample and after three minutes recovery at room temperature (RT), then samples were centrifuged at 12,000 g for 15 minutes at 4° C. After that, the chloroform-RNA phase was collected and an appropriate quantity of isopropanol was added for each sample. After 10 minutes recovery at RT, samples were centrifuged at 12,000 g for 10 minutes 4°C. Then, the pelleted RNA was washed with absolute ethanol and centrifuged at 12,000 g for 5 minutes 4°C. After ethanol removal, the isolated RNA was suspended in nuclease free water and quantified spectrophotometrically by NanoDrop 2000 (Thermofisher Scientific). A total amount of 200 ng of RNA per sample was retro-transcribed in cDNA in a 20 μl reaction using the High Capacity cDNA Reverse Transcription Kit (Applied Biosystems, Thermofisher). Then, a Real Time Polymerase Chain Reaction (RT-PCR) was performed on 5 ng cDNA samples in duplicate, according to the chemistry of Power SYBR® Green PCR Master Mix (Applied Biosystems, Thermofisher), using specific oligonucleotide primers (Supplementary Table 1) and ran by StepOne Real Time System (Applied Biosystems, Thermofisher). Comparative ΔΔCt method was used to calculate the relative expression of genes of interest normalized to the house-keeping gene expression Actinβ. The hepatic biopsies collected at T1 were used as reference samples for the quantitative analysis. Data were expressed as Relative Quantification (RQ) mean ± SEM. One way or two way Anova were performed as statistical analysis, with Tukey’s multiple comparison test. A p-value <0.05 was considered as statistical significant. All analyses were performed using GraphPad Prism 6 software (GraphPad Software, San Diego, California USA, www.graphpad.com).

**Hepatic tissue succinate**

Succinate level was measured on hepatic biopsies using the Succinate Colorimetric Assay Kit (K649, Biovision Incorporated, Milpitas, CA, USA). Briefly, an equal amount of each biopsy was suspended in ice cold Succinate Assay Buffer (K649-1, Biovision Incorporated, Milpitas, CA, USA) together with 0.5 mm size zirconium beads, then homogenized by a Bullet Blender (Next Advance, Inc), at a speed of 8 rpm for 3 minutes at 4°C. Proteins were collected from the homogenized tissue by centrifugations at 12,000 g for 15 min at 4°C. Then, sample was analyzed by the Succinate Colorimetric Assay Kit, according to the manufacturer’s instructions, and the optical density (OD) at 450 nm was read by iMark™ Microplate Absorbance Reader (Bio-Rad, Hercules, CA, USA). Succinate level was normalized towards the T1 biopsy and expressed as Arbitrary Unit (A.U.) mean ± SEM. The Wilcoxon matched-pairs signed rank test was performed between columns with two-sided option. A p-value <0.05 was considered as statistical significant. All analyses were performed using GraphPad Prism 6 software (GraphPad Software, San Diego, California USA, [www.graphpad.com](http://www.graphpad.com)).

**Supplementary table 1:** Forward and reverse oligonucleotides sequences of RT-PCR primers.

| **Gene** | **Forward primer (5’→3’)** | **Reverse primer (3’→5’)** |
| --- | --- | --- |
| ACTβ *(Actin β)* | TGAAGATCAAGATCATTGCTCCTC | CACATCTGCTGGAAGGTGGAC |
| CXCL12  *(CXC motif chemokine 12)* | CACTCCAAACTGTGCCCTTCA | TAGCTTCGGGTCAATGCACA |
| ICAM1  *(Intercellular adhesion molecule 1)* | CATCTACAGCTTTCCGGCG | CTGGGCTGGAACCCCATT |
| IL-6  *(Interleukin-6)* | CTTCAGAACGAATTGACAAACAAATT | TGTTACTCTTGTTACATGTCTCCTTTCTC |
| IL-8  *(Interleukin-8)* | GCTCTGTGTGAAGGTGCAGTTTT | AGTGTGGTCCACTCTCAATCACTCT |
| TLR4  *(Toll-like receptor 4)* | TGGTGTCCCAGCACTTCATCC | ACCAGCACGACTGCTCAGAAA |
| *TNFα*  *(Tumour necrosis factor α)* | CCCAGGGACCTCTCTCTAATCA | GCTTGAGGGTTTGCTACAACATG |

**Supplementary figure 1**. Succinate level of liver biopsies.

Succinate level significantly decreased after reperfusion. No significant differences were observed according to the development of early allograft dysfunction.








| **Supplementary table 2. Microdialysate values stratified by early allograft dysfunction** | | | | |
| --- | --- | --- | --- | --- |
| Value | Overall | No EAD | EAD | p |
|  | 10 | 7 | 3 |  |
| Glucose (μmol/L) |  |  |  |  |
| Backtable | 4300 [3740, 6050] | 3960 [3680, 4850] | 8600 [6150, 8885] | 0.21 |
| 1st hour | 11800 [10475, 12958] | 10820 [9635, 11985] | 12740 [12700, 16580] | 0.14 |
| 2nd hour | 13460 [10520, 19092] | 10730 [10005, 13460] | 21580 [20295, 22050] | 0.03 |
| 3rd hour | 11010 [9595, 13280] | 9595 [8888, 10302] | 15550 [15550, 15550] | 0.22 |
| Lactate (μmol/L) |  |  |  |  |
| Backtable | 1865 [1390, 2335] | 1710 [1135, 2180] | 2320 [1960, 3145] | 0.31 |
| 1st hour | 2775 [2200, 3765] | 2350 [1920, 2950] | 3910 [3445, 4125] | 0.14 |
| 2nd hour | 2890 [2178, 4390] | 2740 [1905, 2890] | 4880 [4050, 5950] | 0.03 |
| 3rd hour | 1550 [1330, 2440] | 1330 [1220, 1440] | 3330 [3330, 3330] | 0.22 |
| Glutamate (μmol/L) |  |  |  |  |
| Backtable | 200 [189, 206] | 201 [189, 205] | 200 [187, 205] | 0.73 |
| 1st hour | 192 [187, 194] | 193 [188, 193] | 191 [182, 195] | 0.73 |
| 2nd hour | 196 [190, 199] | 197 [193, 199] | 179 [177, 190] | 0.21 |
| 3rd hour | 193 [192, 200] | 192 [192, 193] | 207 [207, 207] | 0.22 |
| Pyruvate (μmol/L) |  |  |  |  |
| Backtable | 4 [2, 4] | 3 [2, 4] | 4 [3, 5] | 0.47 |
| 1st hour | 4 [2, 6] | 3 [2, 5] | 4 [4, 5] | 0.29 |
| 2nd hour | 6 [4, 8] | 6 [5, 12] | 5 [4, 6] | 0.65 |
| 3rd hour | 12 [8, 14] | 10 [7, 12] | 12 [12, 12] | 1.00 |
| L/P ratio |  |  |  |  |
| Backtable | 632 [494, 670] | 605 [404, 667] | 662 [621, 731] | 0.43 |
| 1st hour | 909 [568, 1082] | 840 [603, 1180] | 978 [737, 1031] | 0.91 |
| 2nd hour | 571 [215, 884] | 284 [179, 768] | 1220 [811, 1312] | 0.09 |
| 3rd hour | 278 [190, 278] | 190 [147, 234] | 278 [278, 278] | 0.48 |
| Data are expressed as median [interquartile range] and compared using non-parametric Mann-Whitney test. Abbreviations: EAD, early allograft dysfunction; L/P, lactate/pyruvate | | | | |

| **Supplementary table 3.** Normalized perfusate values stratified by EAD | | | | |
| --- | --- | --- | --- | --- |
| Value | Overall | No EAD | EAD | p |
|  | 10 | 7 | 3 |  |
| Glucose (mg/dl) |  |  |  |  |
| 30’ | 193 [174, 212] | 194 [171, 224] | 191.2 [182, 203] | 0.91 |
| 60’ | 198 [189, 223] | 194 [180, 229] | 198.8 [198, 214] | 0.57 |
| 90’ | 204 [189, 225] | 194 [185, 232] | 208.3 [206, 219] | 0.31 |
| 120’ | 226 [210, 244] | 210 [209, 233] | 240.7 [240, 240] | 0.65 |
| Lactate (mmol/L) |  |  |  |  |
| 30’ | 3.4 [3.0, 3.9] | 3.4 [2.8, 3.7] | 3.9 [3.6, 4.1] | 0.43 |
| 60’ | 3.3 [3.0, 3.7] | 3.1 [2.6, 3.3] | 4.1 [3.7, 4.1] | 0.03 |
| 90’ | 3.3 [2.9, 3.5] | 3.3 [2.6, 3.4] | 4.2 [3.7, 4.4] | 0.14 |
| 120’ | 2.8 [2.4, 3.3] | 2.5 [2.3, 3.3] | 3.1 [3.1, 3.1] | 0.65 |
| Glutamate (μmol/L)* |  |  |  |  |
| 30’ | 214 [181, 261] | 233.1 [191, 296] | 202.6 [139, 211] | 0.21 |
| 60’ | 222 [202, 275] | 241.3 [211, 305] | 199.5 [150, 214] | 0.14 |
| 90’ | 224 [203, 267] | 249.6 [210, 317] | 196.2 [146, 214] | 0.09 |
| 120’ | 224 [221, 275] | 275.3 [249, 324] | 163.1 [134, 191] | 0.08 |
| Pyruvate (μmol/L)* |  |  |  |  |
| 30’ | 9 [6, 16] | 12.8 [8, 18] | 5 [3, 6] | 0.09 |
| 60’ | 12 [10, 20] | 12.0 [10, 21] | 10 [7, 16] | 0.57 |
| 90’ | 18 [8, 23] | 22.3 [17, 24] | 4 [2, 7] | 0.03 |
| 120’ | 15 [15, 19] | 19.0 [17, 24] | 8 [5, 11] | 0.08 |
| L/P ratio* |  |  |  |  |
| 30’ | 450 [241, 488] | 269.3 [188, 450] | 625 [559, 1612] | 0.02 |
| 60’ | 241 [159, 365] | 240 [154, 243] | 404 [277, 661] | 0.43 |
| 90’ | 156 [134, 408] | 146 [127, 155] | 881 [594, 3165] | 0.03 |
| 120’ | 170 [116, 226] | 131 [100, 203] | 209 [209, 209] | 0.65 |
| *Perfusate values of glutamate and pyruvate needs to be interpreted with caution as they were constantly close to the low (pyruvate) or high (glutamate) limit of determination of the analyzer. Data are expressed as median [interquartile range] and compared using non-parametric Mann-Whitney test. Abbreviations: EAD, early allograft dysfunction; L/P, lactate/pyruvate | | | | |

| **Supplementary table 4.** Flavin mononucleotide values (RFU) stratified by EAD | | | | |
| --- | --- | --- | --- | --- |
| Value | Overall | No EAD | EAD | p |
|  | 10 | 7 | 3 |  |
| Perfusate |  |  |  |  |
| 30’ | 11701  [6945, 21356] | 9588  [6606, 14146] | 28830  [17764, 31675] | 0.21 |
| 60’ | 13175  [7843, 23158] | 10537  [7388, 17318] | 25362  [16638, 30078] | 0.14 |
| 90’ | 13140  [5477, 23268] | 11548  [4562, 17313] | 32265  [20056, 32267] | 0.14 |
| 120’ | 10629  [8600, 20939] | 8600  [7553, 14769] | 25311  [17970, 32652] | 0.25 |
| ﻿150’ | 11343  [8908, 16324] | 13889  [10181, 17597] | 11343  [11343, 11343] | 1.00 |
| 180’ | 6326  [6326, 6326] | 6326  [6326, 6326] | NA  [NA, NA] | NA |
| Perfusate (normalized to liver weight) | | | | |
| 30’ | 8543  [6177, 12084] | 8174  [6827, 9740] | 12590  [8528, 13486] | 0.43 |
| 60’ | 9566  [5808, 12946] | 8929  [6329, 11971] | 10567  [7922, 12881] | 0.43 |
| 90’ | 9646  [5048, 13815] | 9505  [4484, 11863] | 13444  [9338, 13767] | 0.43 |
| 120’ | 7288  [7086, 15284] | 7288  [6897, 11286] | 11875  [9481, 14269] | 0.56 |
| 150’ | 7562  [7018, 11556] | 11012  [8742, 13281] | 7562  [7562, 7562] | 1.00 |
| 180’ | 6326  [6326, 6326] | 6326  [6326, 6326] | NA  [NA, NA] | NA |
| Microdialysate |  |  |  |  |
| Backtable | 4964  [4706, 5686] | 4978  [4316, 5834] | 4951  [4890, 5113] | 0.91 |
| 60’ | 3482  [3218, 3596] | 3449  [3142, 3543] | 3604  [3408, 4000] | 0.43 |
| 120’ | 3456  [3222, 3670] | 3456  [3261, 3665] | 3430  [3307, 3552] | 1.00 |
| ﻿180’ | 3205  [2992, 3450] | 3236  [3008, 3465] | 3205  [3205, 3205] | 1.00 |
| Data are expressed as median [interquartile range] and compared using non-parametric Mann-Whitney test. Abbreviations: RFU, relative fluorescing units; EAD, early allograft dysfunction; | | | | |
